# Association between Sleep Patterns and Health in Families with Exceptional Longevity

**DOI:** 10.3389/fmed.2017.00214

**Published:** 2017-12-08

**Authors:** Lavy Klein, Tina Gao, Nir Barzilai, Sofiya Milman

**Affiliations:** ^1^Department of Geriatrics, Shoham Medical Center, Pardes-Hanna, The Technion-Israel Institute of Technology, Haifa, Israel; ^2^Institute for Aging Research, Department of Medicine, Albert Einstein College of Medicine, Bronx, NY, United States; ^3^Department of Genetics, Albert Einstein College of Medicine, Bronx, NY, United States

**Keywords:** centenarians, longevity, aging, sleep, nap, age-related diseases

## Abstract

**Background:**

Sleep patterns such as longer sleep duration or napping are associated with poor health outcomes. Although centenarians and their offspring demonstrate a delayed onset of age-related diseases, it is not known whether they have healthier sleep patterns or are protected against the negative effects of sleep disturbances.

**Methods:**

Data on sleep patterns and health history were collected from Ashkenazi Jewish subjects of the Longevity Genes Project using standardized questionnaires. Participants included individuals with exceptional longevity (centenarians) with preserved cognition (*n* = 348, median age 97 years), their offspring (*n* = 513, median age 69 years), and controls (*n* = 199) age-matched to the offspring. Centenarians reported on their sleep patterns at age 70, while the offspring and controls on their current sleep patterns. Biochemical parameters were measured at baseline. Models were adjusted for age, sex, BMI, and use of sleep medication.

**Results:**

The offspring and controls reported similar sleep patterns, with 33% sleeping ≥8 h and 17% napping in each group. At age 70, centenarians were more likely to have slept ≥8 h (55%) and to have napped (28%) compared with offspring and controls, *p* < 0.01. Among centenarians, no association was noted between sleep patterns and health outcomes. Sleeping for ≥8 h was associated with lower high-density lipoprotein cholesterol levels in the offspring and controls, and with insulin resistance in the offspring, but not with diabetes. Napping was associated with insulin resistance among the controls (*p* < 0.01), but not the offspring. Controls, but not offspring, who napped were 2.79 times more likely to have one or more of the following diseases: hypertension, myocardial infarction, stroke, or diabetes (OR 2.79, 95% CI 1.08–7.21, *p* = 0.04).

**Conclusion:**

Despite being more likely to exhibit risky sleep patterns at age 70 compared with the offspring and controls, the centenarians were protected from age-related morbidities. The offspring of centenarians did exhibit metabolic disturbances in association with less healthy sleep patterns; however, unlike the controls, they were much less likely to manifest age-related diseases. This suggests that offspring may have inherited resilience genotypes from their centenarian parents that protect them against the harmful effects of sleep disturbances.

## Introduction

Sleep is the restorative phase of the daily arousal-sleep cycle. A circadian clock, which includes the suprachiasmatic nucleus (SCN) and related structures in the brain, is genetically regulated through identified circadian genes (1, 2), and controls sleep patterns (3). The circadian genes also regulate the metabolic and hormonal diurnal and nocturnal fluctuations of the organism (4, 5). Peripheral circadian clocks in organs such as the liver (6) and the kidney (7) are synchronized with the main SCN clock. Aging of the circadian clock system is associated with sleep disorders in the elderly (8–10) and with characteristic age-related changes in glucose and lipid metabolism (4, 11, 12). Thus, sleep disorders and metabolic changes are regulated *via* a common apparatus, the circadian clock. This may explain the link between sleep disorders and metabolic diseases like diabetes and obesity. Like other genetically determined traits, sleep pattern is subjected to changes in circadian-gene expression that may be influenced by epigenetic alterations that result from aging (13, 14).

Sleep patterns change throughout the lifespan. Predictable changes in sleep quality that frequently affect older individuals include reduced hours of nighttime sleep, sleep fragmentation, and daytime napping (15). Metabolic function in humans is linked to sleep duration and sleep quality. Glycemic control is regulated by the sleep-arousal cycle (16) and sleep loss has been associated with insulin resistance and Type 2 diabetes mellitus (T2DM) (17). Experimentally controlled circadian rhythm disruption in diabetes-prone transgenic rats has accelerated development of diabetes through pancreatic cell dysfunction (18). Cortisol, growth hormone, leptin, and ghrelin levels are also modulated by sleep duration and quality (19). Preservation of regular sleep-wake patterns has been associated with higher high-density lipoprotein (HDL) cholesterol and lower triglyceride levels in the elderly (20). The impact of sleep patterns on mortality has also been widely investigated in longitudinal studies. In a recent meta-analysis of 27 cohort studies, long and short sleep duration were associated with increased all-cause mortality in the elderly (21).

Whether sleep pattern is primarily a genetically determined phenotype or a lifestyle habit remains open to debate. However, there are studies which show that sleep disorders are heritable and that genetic factors play a substantial role in the pathophysiology of sleep disorders (22–24). Exceptional longevity is also an inherited trait and first-degree relatives of centenarians are 8–17 times more likely to achieve longevity themselves (25). Centenarians delay the onset of most age-related diseases and exhibit unique biological phenotypes (26–29), which are often inherited by their offspring (30–32). This can explain why centenarian offspring stay healthier and live longer (33–35). With the knowledge that sleep patterns are associated with metabolic conditions, we conducted a study looking at whether reported sleep quality, sleep duration, and daytime napping were associated with health outcomes in individuals with exceptional longevity, their offspring, and offspring of parents with usual life expectancy. The hypothesis underlying our study was that centenarians and their offspring are protected from age-related sleep disturbances or the negative health impact of sleep disturbances.

## Materials and Methods

### Study Population

Participants from the cross-sectional Longevity Genes Project (LGP) that was initiated in 1998 were the subjects of this study. Detailed description of the LGP study is available elsewhere (31, 36). In brief, LGP recruited individuals from the Northeastern United States age 95 and older who were living independently at the age of 95, which served as a reflection of general good health (centenarians). In addition, LGP enrolled the offspring of centenarians and controls, most of who were the spouses of the offspring but did not have a centenarian parent. The ages of the centenarians were verified with government issued identification. All of the study subjects were Ashkenazi Jewish, defined by having all four grandparents who were Ashkenazi Jewish. Participants with cognitive impairment, which was defined by Mini-Mental State Examination (MMSE) score ≤22 (37, 38) or blind MMSE <16 (39) were excluded from this analysis because the administered sleep questionnaire relied on self-report. The study was approved by the Institutional Review Board at the Albert Einstein College of Medicine. Written informed consent was obtained from all the study participants in accordance with the Declaration of Helsinki.

### Sleep Pattern and Health Outcomes

Self-reported sleep patterns and health outcome data from the LGP were analyzed. Due to several revisions of the sleep questionnaire throughout the duration of the study, three versions of the questionnaires existed. All three versions have addressed the following sleep patterns at age 70 for centenarians: sleep duration in a 24 hour period, regular daily napping behavior, and presence of sleep problems. A subset of centenarians also reported on their current sleep patterns at the age of enrollment (*n* = 43). Offspring and controls reported on their current sleep pattern since not all of them have reached age 70. Sleep medication usage at the time of enrollment was reported by all participants. Participants who reported using sleep medications were considered as having a sleep problem.

History of the following diseases was self-reported by all participants: myocardial infarction (MI), hypertension (HTN), stroke or transient ischemic attack (TIA), and diabetes. Physical assessments included measurements of height and weight. Body mass index (BMI) was calculated according to the following formula: BMI = mass (kg)/height (m)^2^. The following metabolic parameters were measured at the time of enrollment among the offspring and controls at the Montefiore Medical Center clinical laboratories and the Biomarker Analytic Research Core at the Albert Einstein College of Medicine: insulin (excluding participants using insulin or diabetes medications), glucose, HDL cholesterol, high-sensitivity C-reactive protein (CRP), and creatinine. Insulin resistance was quantified by the homeostatic model assessment, HOMA-IR (40), and the glomerular filtration rate (eGFR) was calculated (41). Insulin-like growth factor 1 (IGF-1) and testosterone (in males only) were measured using liquid chromatography/mass spectrometry in previously frozen serum at Quest Diagnostics Nichols Institute laboratories, San Juan Capistrano, CA, USA.

### Statistical Analysis

Descriptive statistics for sleep pattern and health outcomes were reported. Daily sleep duration was dichotomized into <8 and ≥8 h. Napping behavior, sleep problems, and daily sleep medication usage were coded as “yes” or “no.” In addition to examining history of MI, HTN, stroke or TIA, and diabetes individually, a binary comorbidity index was also created for the aforementioned conditions. Participants who reported having one or more of these diseases would receive a score of one, whereas a score of zero was recorded if none of these diseases were reported. Mann–Whitney, chi-square, and Student’s *t*-tests were applied to evaluate significant differences of sleep pattern and health outcomes between sub-cohorts.

For centenarians, a multivariable logistic regression model adjusted for age and sex was applied to examine the associations between each sleep pattern at age 70 (hours of sleep, napping behavior, and sleep problem) and disease history (MI, HTN, stroke/TIA, diabetes, and the comorbidity index). For offspring and controls, two adjusted multivariable logistic regression models were built to determine the associations between each sleep pattern and aforementioned diseases, and two adjusted linear regression models were used for physical and metabolic parameters. For both logistic and linear regressions, Model 1 was adjusted for age, sex, and BMI, while Model 2 was adjusted for the same parameters as Model 1 and additionally for use of sleep medication. CRP level was log-transformed and analysis involving IGF-1 was stratified by sex due to its sex-dependent characteristics. eGFR was only adjusted for BMI in Model 1 and additionally for sleeping medication usage in Model 2, because age and sex were already incorporated into its calculation. Odds ratios with 95% confidence intervals (CI) and beta coefficients with 95% CI were reported for all logistic regression and linear regression models, respectively. A *p*-value of <0.05 was considered statistically significant. STATA version 12 (College Station, TX, USA) was used for statistical analyses.

## Results

As shown in Table 1, the median age of centenarians (*n* = 348) at enrollment was 96.8 [interquartile range (IQR) 95.5–99.3] years, of offspring (*n* = 513) 69.3 (IQR 63.1–74.4) years, and of controls (*n* = 199) 70.2 (IQR 63.2–76.5) years. The majority of centenarians were female (69%). Centenarians were significantly more likely to have slept ≥8 h per day and nap regularly at age 70 compared with offspring and controls (*p* < 0.01). Although there was no significant difference between the hours of sleep at current age and at age 70 for centenarians, centenarians were more likely to nap (45.2 vs. 28.1%, *p* < 0.05) and report having sleeping problems (80.8 vs. 28.5%, *p* < 0.01) at current age than at age 70.

**Table 1:** Demographic characteristics and sleep patterns at enrollment and at age 70.

	Centenarians (*n* = 348)		Offspring (*n* = 513)	Controls (*n* = 199)	*p*-Value^a*^^,^^b*^^,^^a^^,^^b^^,^^c^^,^^d^	
Median age at enrollment (interquartile range), years	96.8 (95.5–99.3)		69.3 (63.1–74.4)	70.2 (63.2–76.5)	a*: <0.01 b*: <0.01	c: 0.18
Female %, (*n*)	69 (240)		52.8 (271)	52.8 (105)	a*: <0.01 b*: <0.01	c: 0.98

**Sleep patterns**	**At age 70**	**At enrollment**	**At enrollment**			

Mean duration of sleep ± SD, h (*n*)	7.5 ± 1.3 (263)	7.7 ± 2.5 (32)	7.1 ± 1.1 (461)	7.1 ± 1.0 (178)	a: <0.01 b: <0.01	c: 0.96 d: 0.67
≥8 h of sleep %, (*n*)	55.1 (145)	50 (16)	32.8 (151)	33.2 (59)	a: <0.01 b: <0.01	c: 0.93 d: 0.58
Napped %, (*n*)	28.1 (83)	45.2 (14)	17.7 (80)	17.1 (30)	a: <0.01 b: <0.01	c: 0.85 d: <0.05
Sleeping problem present %, (*n*)	28.5 (80)	80.8 (59)	32.8 (146)	31 (53)	a: 0.22 b: 0.57	c: 0.67 d: <0.01
Current sleeping pill usage %, (*n*)	–	25.1 (50)	12.2 (41)	15.9 (18)	c: 0.31	

^a*^p-Value obtained from comparison between offspring and centenarians at enrollment.

^b*^p-Value obtained from comparison between controls and centenarians at enrollment.

*^a^p-Value obtained from comparison between offspring and centenarians at age 70*.

*^b^p-Value obtained from comparison between controls and centenarians at age 70*.

*^c^p-Value obtained from comparison between offspring and controls*.

*^d^p-Value obtained from comparison between centenarians at age 70 and centenarians at enrollment*.

Among centenarians, those who had ≥8 h of sleep at age 70 had 0.27 (95% CI 0.14–0.49, *p* < 0.01) times the odds of reporting sleep problems, whereas those who napped regularly at age 70 had 2.85 (95% CI 1.6–5.05, *p* < 0.01) times the odds of reporting sleep problems, after adjusting for age and sex (Table 2). There were no significant associations between sleep patterns at age 70 and any disease history in centenarians assessed at the time of enrollment (Table 2).

**Table 2:** Centenarians’ sleep patterns at age 70 and disease history at enrollment.

	Sleep hours				Nap				Sleep problems			
	<8 h (*n* = 118)	≥8 h (*n* = 145)	*p*-Value^a^	OR (95% CI)^b^	Yes (*n* = 83)	No (*n* = 212)	*p*-Value^c^	OR (95% CI)^d^	Yes (*n* = 80)	No (*n* = 201)	*p*-Value^e^	OR (95% CI)^f^
Median age (IQR), years	97.3 (95.6–100.3)	96.5 (95.5–98.8)	0.11		96.5 (95.7–100.1)	96.9 (95.4–99.2)	0.49		97.1 (95.9–100.2)	96.8 (95.5–99.4)	0.18	
Female %, (*n*)	72 (85)	64.1 (93)	0.17		68.7 (57)	70.8 (150)	0.73		75 (60)	68.2 (137)	0.26	

**Sleep patterns at age 70**												

Napped %, (*n*)	25.7 (29)	31.2 (44)	0.33	1.37 (0.78–2.4), *p* = 0.27	–	–	–	–	–	–	–	–
Sleep problems %, (*n*)	41.9 (44)	15.8 (22)	<0.01	0.27 (0.14–0.49), *p* < 0.01	42.9 (33)	20.7 (40)	<0.01	2.85 (1.6–5.05), *p* < 0.01	–	–	–	–

**Disease history**												

MI %, (*n*)	15.2 (16)	14.3 (18)	0.84	0.96 (0.46–2.01), *p* = 0.92	17.1 (13)	15.1 (28)	0.68	1.12 (0.54–2.31), *p* = 0.76	20 (15)	14.9 (26)	0.32	1.42 (0.7–2.89), *p* = 0.33
HTN %, (*n*)	59.3 (67)	59.4 (79)	0.99	1.04 (0.62–1.76), *p* = 0.87	55.1 (43)	60.5 (121)	0.41	0.82 (0.48–1.4), *p* = 0.47	59.7 (46)	56.7 (106)	0.65	1.07 (0.62–1.86), *p* = 0.81
Stroke/TIA %, (*n*)	22.6 (26)	21.1 (30)	0.78	0.92 (0.5–1.67), *p* = 0.77	22.2 (18)	20.8 (43)	0.79	1.13 (0.6–2.11), *p* = 0.71	19 (15)	23 (45)	0.47	0.77 (0.4–1.49), *p* = 0.43
Diabetes %, (*n*)	4.7 (5)	7.3 (9)	0.42	1.38 (0.44–4.36), *p* = 0.58	6.6 (5)	7 (13)	0.91	0.99 (0.34–2.9), *p* = 0.98	4.1 (3)	7.4 (13)	0.32	0.54 (0.15–1.97), *p* = 0.35
Positive comorbidity index %, (*n*)	76.4 (84)	70.2 (92)	0.29	0.74 (0.41–1.35), *p* = 0.33	70.1 (54)	74 (145)	0.52	0.86 (0.47–1.56), *p* = 0.62	70.7 (53)	72.6 (135)	0.76	0.86 (0.47–1.57), *p* = 0.62

*^a^p-Value comparing centenarians with <8 vs. ≥8 h of sleep*.

*^b^Age and sex adjusted OR of disease for those with ≥8 h of sleep compared with those with <8 h of sleep*.

*^c^p-Value comparing centenarians who do and do not nap*.

*^d^Age and sex adjusted OR of disease for those who nap compared with those who do not nap*.

*^e^p-Value comparing centenarians with and without sleep problems*.

*^f^Age and sex adjusted OR of disease for those with sleep problems compared with those without sleep problems*.

As shown in Table 3, the offspring who had ≥8 h of sleep had 1.96 (95% CI 1.06–3.61, *p* = 0.03) times the odds of napping regularly in a model adjusted for age, sex, BMI and sleep medication use (Model 2), and had 0.39 (95% CI 0.24–0.63, *p* < 0.01) times the odds of reporting sleep problems in a model adjusted for age, sex, and BMI (Model 1). Offspring who slept ≥8 h also had statistically significantly higher levels of insulin, insulin resistance (HOMA), as well as marginally higher glucose level. Furthermore, they had lower eGFR and marginally lower HDL cholesterol level than their counterparts who slept <8 h. Similar to offspring, controls who slept ≥8 h were less likely to report sleep problems, OR = 0.35 (95% CI 0.16–0.76, *p* < 0.01) and were found to have significantly lower level of HDL cholesterol than their counterparts who had <8 h of sleep (Table 3) (Model 2). However, no statistically significant associations were noted between sleep duration and any of the diseases among the offspring or controls.

**Table 3:** Associations between sleep duration and disease or biochemical profile for offspring and controls.

	Offspring					Controls				
	Sleep hours		*p*-Value^a^	Model 1: OR (95% CI)^b^	Model 2: OR (95% CI)^c^	Sleep hours		*p*-Value^a^	Model 1: OR (95% CI)^b^	Model 2: OR (95% CI)^c^
	<8 h (*n* = 310)	≥8 h (*n* = 151)				<8 h (*n* = 119)	≥8 h (*n* = 59)			
Median age (IQR), years	69 (62.2–74.4)	70.3 (64.6–75.8)	0.02			70.3 (63–76.3)	70 (65.2–77.5)	0.34		
Female %, (*n*)	51.9 (161)	58.3 (88)	0.2			50.4 (60)	54.2 (32)	0.63		

**Sleep patterns**										

Napped %, (*n*)	15.3 (46)	23 (34)	<0.05	1.66 (0.99–2.78), *p* = 0.05	1.96 (1.06–3.61), *p* = 0.03	12.9 (15)	23.7 (14)	0.07	2.1 (0.88–5), *p* = 0.09	2.66 (0.86–8.24), *p* = 0.09
Sleep problem %, (*n*)	38.1 (114)	19.7 (28)	<0.01	0.39 (0.24–0.63), *p* < 0.01	–	37.8 (42)	18.6 (11)	0.01	0.35 (0.16–0.76), *p* < 0.01	–
Sleeping pill usage %, (*n*)	11.8 (27)	11.9 (12)	0.99	0.95 (0.45–1.99), *p* = 0.89	–	17.1 (13)	13.5 (5)	0.62	0.75 (0.24–2.31), *p* = 0.62	–

**Disease history**										

MI %, (*n*)	4.3 (13)	4.9 (7)	0.78	0.93 (0.34–2.51), *p* = 0.88	0.7 (0.18–2.72), *p* = 0.61	7.8 (9)	8.6 (5)	0.84	1.04 (0.31–3.57), *p* = 0.94	0.63 (0.07–5.43), *p* = 0.67
HTN %, (*n*)	34.8 (103)	44.7 (63)	<0.05	1.48 (0.94–2.32), *p* = 0.09	1.4 (0.79–2.49), *p* = 0.25	37.4 (43)	49.1 (27)	0.15	1.58 (0.8–3.12), *p* = 0.19	2.12 (0.86–5.21), *p* = 0.1
Stroke/TIA %, (*n*)	1 (3)	2.1 (3)	0.33	1.51 (0.27–8.43), *p* = 0.64	1.61 (0.2–13.28), *p* = 0.66	0.9 (1)	7.1 (4)	0.02	8.01 (0.82–78.3), *p* = 0.07	Omitted due to small sample size
Diabetes %, (*n*)	6.9 (21)	7.7 (11)	0.77	1.15 (0.5–2.62), *p* = 0.74	1.44 (0.5–4.17), *p* = 0.5	8.6 (10)	8.5 (5)	0.99	0.84 (0.25–2.84), *p* = 0.78	1.21 (0.27–5.39), *p* = 0.8
Positive comorbidity index %, (*n*)	40.7 (118)	49.3 (69)	0.09	1.41 (0.89–2.23), *p* = 0.14	1.32 (0.74–2.34), *p* = 0.35	47.8 (54)	57.4 (31)	0.25	1.37 (0.69–2.72), *p* = 0.38	2.1 (0.84–5.24), *p* = 0.11

	**Offspring**					**Controls**				
	**Median (IQR)**		*****p***-Value^a^**	**Model 1: β-coef (95% CI)^d^**	**Model 2: β-coef (95% CI)^e^**	**Median (IQR)**		***p*-Value^a^**	**Model 1: β-coef (95% CI)^d^**	**Model 2: β-coef (95% CI)^e^**
	**<8 h (***n*** = 310)**	**≥8 h (***n*** = 151)**				**<8 h (***n*** = 119)**	**≥8 h (***n*** = 59)**			

BMI kg/m^2^, (*n*)	25.2 (23.2–28.4), (309)	25.3 (23.2–28.2), (149)	0.95	−0.25 (−1.07 to 0.57), *p* = 0.55	−0.44 (−1.44 to 0.56), *p* = 0.39	25.3 (23.5–28.3), (118)	24.9 (22.6–27.9), (58)	0.66	−0.12 (−1.57 to 1.33), *p* = 0.87	−0.08 (−2.16 to 1.99), *p* = 0.94

**Biochemical measures**										

Insulin mU/L, (*n*)	11.8 (6.9–26.7), (150)	14.2 (8.7–24.9), (68)	0.13	7.53 (0.66–14.4), *p* = 0.03	5.25 (−1.25 to 11.76), *p* = 0.11	16.1 (10.6–27.1), (58)	13.4 (9.4–21.6), (27)	0.32	−3.06 (−11.18 to 5.06), *p* = 0.46	2.81 (−7.76 to 13.39), *p* = 0.6
Glucose mg/dL, (*n*)	84 (74–96), (297)	86 (76–98), (147)	0.32	2.29 (−3.7 to 8.28), *p* = 0.45	6.97 (−0.62 to 14.56), *p* = 0.07	86 (76–96), (115)	85 (76–98), (54)	0.74	3.73 (−5.03 to 12.48), *p* = 0.4	5.21 (−6.19 to 16.62), *p* = 0.37
Insulin resistance HOMA, (*n*)	2.3 (1.3–5.9), (144)	3.1 (1.6–6), (66)	0.06	2.85 (0.48–5.23), *p* = 0.02	2.4 (0.14–4.66), *p* = 0.04	3.5 (2–5.7), (57)	3 (1.9–5), (22)	0.45	−1.3 (−3.48 to 0.88), *p* = 0.24	−0.35 (−2.97 to 2.27), *p* = 0.79
HDL mg/dL, (*n*)	61 (51–74), (305)	59 (47–73), (150)	0.19	−2.71 (−5.94 to 0.52), *p* = 0.1	−3.75 (−7.63 to 0.14), *p* = 0.06	60 (51–72), (117)	60 (49–75), (59)	0.53	−1.66 (−6.23 to 2.9), *p* = 0.47	−5.94 (−11.3 to −0.58), *p* = 0.03
IGF-1 ng/mL, (*n*)	119 (97.5–144), (264)	125 (92–146), (130)	0.86	0.12 (−8.38 to 8.61), *p* = 0.98	4.98 (−5.17 to 15.12), *p* = 0.34	110 (89–145), (79)	123.5 (92.5–146), (44)	0.3	10.31 (−5.79 to 26.41), *p* = 0.21	5.22 (−13.93 to 24.37), *p* = 0.59
CRP^f^ mg/L, (*n*)	0.2 (0.1–0.4), (156)	0.25 (0.1–0.4), (85)	0.43	0.06 (−0.16 to 0.28), *p* = 0.6	0.05 (−0.2 to 0.3), *p* = 0.69	0.3 (0.1–0.5), (59)	0.3 (0.2–0.7), (31)	0.17	0.14 (−0.2 to 0.49), *p* = 0.42	0.21 (−0.19 to 0.61), *p* = 0.3
eGFR^g^ mL/min/1.73 m^2^, (*n*)	75.6 (64.4–88.1), (296)	71.7 (59.4–86.1), (145)	0.04	−3.63 (–6.93 to −0.32), *p* = 0.03	−5 (−8.91 to −1.09), *p* = 0.01	71.4 (62.1–82.9), (116)	73 (60.1–86.2), (55)	0.87	−0.22 (−5.81 to 5.37), *p* = 0.94	0.41 (−6.83 to 7.66), *p* = 0.91
Testosterone^h^ ng/dL, (*n*)	391 (310–500), (129)	362 (302–503), (54)	0.46	−9.93 (−61.64 to 41.78), *p* = 0.71	−20.71 (−81.8 to 40.39), *p* = 0.5	383 (298–500), (37)	323 (290–405), (20)	0.19	−61.57 (−129.63 to 6.49), *p* = 0.08	−63.24 (−149.16 to 22.69), *p* = 0.14

*^a^p-Value comparing those with <8 vs. ≥8 h of sleep*.

*^b^Age, sex, and BMI adjusted OR of disease for those with ≥8 h of sleep compared with those with <8 h of sleep*.

*^c^Age, sex, BMI, and sleep pill adjusted OR of disease for those with ≥8 h of sleep compared with those with <8 h of sleep*.

*^d^Age, sex, and BMI adjusted beta coefficient for ≥8 h of sleep compared with those with <8 h of sleep*.

*^e^Age, sex, BMI, and sleep pill adjusted beta coefficient for ≥8 h of sleep compared with those with <8 h of sleep*.

*^f^Beta coefficients are log transformed*.

*^g^Model 1 is adjusted only for BMI and Model 2 is adjusted only for BMI and sleeping pill usage*.

*^h^Testosterone is reported for males only*.

Regular napping was not associated with self-reported sleep problems, sleeping pill usage, or any of the individual diseases among offspring and controls (Table 4). However, controls who napped regularly had significantly higher odds of having one or more conditions in the comorbidity index after adjusting for age, sex, and BMI (OR = 2.79, 95% CI 1.08–7.21, *p* = 0.04). Offspring who napped regularly had higher level of glucose, but not insulin or HOMA. Regular nappers among the offspring also had lower IGF-1 level than their counterparts who did not nap. However, the IGF-1 level was only significantly lower in male offspring (sex-stratified data not shown in Table 4). On the other hand, controls who napped had higher levels of insulin and insulin resistance, and had lower eGFR. Presence of self-reported sleep problems was not significantly associated with any diseases, physical parameters, or biochemical markers (data not shown).

**Table 4:** Associations between napping behavior and disease or biochemical profile for offspring and controls.

	Offspring					Controls				
	Nap		*p*-Value^a^	Model 1: OR (95% CI)^b^	Model 2: OR (95% CI)^c^	Nap		*p*-Value^a^	Model 1: OR (95% CI)^b^	Model 2: OR (95% CI)^c^
	Yes (*n* = 80)	No (*n* = 372)				Yes (*n* = 30)	No (*n* = 146)			
Median age (IQR), years	72.9 (65.7–77.6)	69.3 (62.9–74.2)	<0.01			75.4 (69.1–81.1)	69.1 (63–75)	<0.01		
Female %, (*n*)	40 (32)	57 (212)	<0.01			43.3 (13)	54.8 (80)	0.25		

**Sleep patterns**										

Sleep problem %, (*n*)	31.6 (24)	32.9 (118)	0.83	0.98 (0.57–1.69), *p* = 0.94	–	26.7 (8)	31.9 (44)	0.58	0.67 (0.27–1.71), *p* = 0.41	–
Sleeping pill usage %, (*n*)	12.3 (7)	12 (32)	0.95	1.08 (0.44–2.65), *p* = 0.87	–	25 (4)	14.6 (14)	0.29	1.93 (0.52–7.18), *p* = 0.33	–

**Disease history**										

MI %, (*n*)	6.4 (5)	4.2 (15)	0.39	1.11 (0.38–3.26), *p* = 0.85	0.82 (0.2–3.35), *p* = 0.78	16.7 (5)	6.3 (9)	0.06	1.49 (0.4–5.52), *p* = 0.55	1.13 (0.13–9.81), *p* = 0.91
HTN %, (*n*)	39.5 (30)	38 (134)	0.81	0.72 (0.41–1.25), *p* = 0.24	0.89 (0.45–1.76), *p* = 0.74	62.1 (18)	36 (50)	<0.01	2.16 (0.91–5.12), *p* = 0.08	2.4 (0.78–7.43), *p* = 0.13
Stroke/TIA %, (*n*)	3.9 (3)	0.8 (3)	0.04	3.22 (0.61–16.88), *p* = 0.17	3.11 (0.38–25.36), *p* = 0.29	10.3 (3)	1.4 (2)	0.01	9.44 (0.85–104.26), *p* = 0.07	Omitted due to small sample size
Diabetes %, (*n*)	11.5 (9)	6.1 (22)	0.09	1.37 (0.58–3.25), *p* = 0.48	2.6 (0.93–7.24), *p* = 0.07	17.2 (5)	6.2 (9)	<0.05	3.55 (0.98–12.8), *p* = 0.05	5.02 (0.9–27.97), *p* = 0.07
Positive comorbidity index %, (*n*)	49.3 (37)	42.7 (148)	0.29	0.85 (0.48–1.48), *p* = 0.56	1.06 (0.54–2.07), *p* = 0.87	75.9 (22)	44.9 (61)	<0.01	2.79 (1.08–7.21), *p* = 0.04	2.8 (0.83–9.37), *p* = 0.1

	**Offspring**					**Controls**				
	**Median (IQR)**		***p*-Value^a^**	**Model 1: β coef (95% CI)^d^**	**Model 2: β coef (95% CI)^e^**	**Median (IQR)**		***p*-Value^a^**	**Model 1: β coef (95% CI)^d^**	**Model 2: β coef (95% CI)^e^**
	**Yes (***n*** = 80)**	**No (***n*** = 372)**				**Yes (***n*** = 30)**	**No (***n*** = 146)**			

BMI kg/m^2^, (*n*)	26.5 (23.7–29.1), (80)	25.1 (23.1–28.2), (369)	0.07	0.81 (−0.21 to 1.83), *p* = 0.12	0.4 (−0.82 to 1.61), *p* = 0.52	25.9 (24.1–28.6), (30)	25.1 (22.7–28.2), (144)	0.13	1.28 (−0.58 to 3.15), *p* = 0.18	2.1 (−0.7 to 4.9), *p* = 0.14

**Biochemical measures**										

Insulin mU/L, (*n*)	16.1 (8.7–38.5), (38)	12 (7–23), (176)	0.16	2.64 (−6.07 to 11.36), *p* = 0.55	−0.19 (−8.32 to 7.94), *p* = 0.96	19 (14.6–49.8), (13)	15.3 (9–22.7), (72)	0.03	15.8 (5.81–25.78), *p* < 0.01	16.8 (−1.19 to 34.78), *p* = 0.07
Glucose mg/dL, (*n*)	87 (75–111), (77)	84 (75–94), (357)	0.08	6.56 (−0.93 to 14.05), *p* = 0.09	11.91 (2.73–21.09), *p* = 0.01	88 (79–102), (30)	83 (75–95), (137)	0.16	0.32 (−10.7 to 11.34), *p* = 0.95	−1.39 (−16.76 to 13.97), *p* = 0.86
Insulin resistance HOMA, (*n*)	3.6 (1.5–8.7), (37)	2.4 (1.3–5.4), (168)	0.09	0.7 (−2.3 to 3.69), *p* = 0.65	0.36 (−2.48 to 3.2), *p* = 0.8	3.7 (2.9–12.1), (13)	3.3 (1.7–4.8), (66)	<0.05	3.72 (1.18–6.27), *p* < 0.01	6.61 (2.67–10.55), *p* < 0.01
HDL mg/dL, (*n*)	54 (46–66), (79)	62 (51–76), (367)	<0.01	−2.75 (−6.79 to 1.28), *p* = 0.18	−3.49 (−8.22 to 1.24), *p* = 0.15	54 (47–66), (30)	61 (52–75), (144)	<0.05	−3.79 (−9.68 to 2.09), *p* = 0.21	−4.26 (−11.71 to 3.2), *p* = 0.26
IGF-1 ng/mL, (*n*)	110 (82–138), (67)	124 (98–145.5), (316)	0.03	−13.58 (−24.13 to −3.03), *p* = 0.01	−13.47 (−26.09 to −0.84), *p* = 0.04	105 (74–136), (20)	118 (94–145), (101)	0.28	−9.31 (−30.52 to 11.9), *p* = 0.39	4.13 (−23.09 to 31.34), *p* = 0.76
CRP^f^ mg/L, (*n*)	0.3 (0.1–0.5), (44)	0.2 (0.1–0.4), (193)	0.37	0.06 (−0.21 to 0.34), *p* = 0.66	0.09 (−0.21 to 0.39), *p* = 0.57	0.5 (0.2–0.6), (17)	0.3 (0.2–0.5), (71)	0.21	−0.004 (−0.44 to 0.43), *p* = 0.99	0.12 (−0.38 to 0.62), *p* = 0.64
eGFR^g^ mL/min/1.73 m^2^, (*n*)	73 (59.6–85.8), (77)	75.6 (62.7–88.5), (354)	0.20	−1.95 (−6.04 to 2.15), *p* = 0.35	−3.04 (−7.72 to 1.64), *p* = 0.2	68.1 (55.9–76), (30)	73.3 (61.8–86.9), (139)	0.05	−7.08 (−13.83 to −0.33), *p* = 0.04	−9.74 (−19.18 to −0.31), *p* = 0.04
Testosterone^h^ ng/dL, (*n*)	403.5 (325–532), (40)	371.5 (300–491), (138)	0.40	46.89 (−8.76 to 102.55), *p* = 0.1	26.01 (−37.47 to 89.49), *p* = 0.42	349 (277–378), (10)	358 (298–500), (45)	0.43	−20.41 (−113.12 to 72.3), *p* = 0.66	−52.64 (−168.58 to 63.3), *p* = 0.36

*^a^p-Value comparing those who nap and do not nap*.

*^b^Age, sex, and BMI adjusted OR of disease for those who nap compared with those who do not nap*.

*^c^Age, sex, BMI, and sleep pill adjusted OR of disease for those who nap compared with those who do not nap*.

*^d^Age, sex, and BMI adjusted beta coefficient for those who nap compared with those who do not nap*.

*^e^Age, sex, BMI, and sleep pill adjusted beta coefficient for those who nap compared with those who do not nap*.

*^f^Beta coefficients are log transformed*.

*^g^Model 1 is adjusted only for BMI and Model 2 is adjusted only for BMI and sleeping pill usage*.

*^h^Testosterone is reported for males only*.

## Discussion

Favorable sleep patterns have been associated with decreased risk of disease and mortality in the general population. Interestingly, in this study individuals with exceptional longevity did not report more favorable sleep patterns at age 70 compared with similarly aged controls, who were not genetically enriched for longevity. Furthermore, the sleep patterns between the offspring of centenarians and controls did not differ, suggesting that genetic predisposition to longevity is not dependent on healthy sleep patterns. Despite demonstrating similar sleep patterns, the offspring of centenarians with unhealthy sleep patterns were significantly less likely to manifest age-related diseases compared with controls with unhealthy sleep patterns. This finding suggests that centenarians and their offspring possess protective genes that make them resilient to the adverse effects of unfavorable sleep patterns.

Other clinical studies on sleep quality among centenarians have reported various results, ranging from reports of overall good sleep (42) to reports of sleeping problems among the majority of the participants (43). The majority of centenarians in our study reported sleeping problems at enrollment, but had a similar prevalence of sleep problems at age 70 compared with offspring and controls. However, there were more centenarians who slept ≥8 h and who had taken daytime naps at age 70 than offspring and controls. Although the centenarians did not report healthy sleep patterns at age 70, prolonged sleep and daytime napping at that age were not associated with diabetes or cardiovascular diseases later in life in this group. Furthermore, all the centenarians in this study had preserved cognition. These findings suggest that among individuals with genetic predisposition to exceptional longevity, sleep disturbances do not negatively impact health outcomes or survival.

The association between sleep duration and survival has been widely investigated. The National Health and Nutrition Examination Survey I (NHANES I) found a U-shaped relationship between sleep duration and mortality in elderly subjects (44). In a subsequent meta-analysis of 13 independent cohort studies, a U-shaped relationship between sleep duration and mortality has been confirmed (45). The Bambui Health and Aging Study, on the other hand, found a linear correlation between sleep duration and mortality (46). The largest report with more than 1 million participants aged 30–102 years concluded that 7 h of sleep was associated with the lowest mortality risk (47). In fact, prolonged, rather than short sleep duration, was more consistently associated with higher mortality in older populations (48–50). Longer duration of sleep was associated not only with overall mortality but also with mortality from cardiovascular diseases (21) and from dementia (51, 52). A number of possible mechanisms have been suggested to explain this relationship, including fatigue, cardiorespiratory and underlying diseases, as well as impaired immune function (53). However, frailty and chronic inflammation have been found to be insignificant factors in long-sleep-associated mortality in Taiwan (48). Considering the epidemiological evidence for association of longer sleep duration and mortality, some have even recommended sleep restriction for the elderly (54). However, there is no evidence that sleep restriction would be helpful, both because there is a lack of randomized clinical trials investigating this question and because longer sleep duration may not be the causative factor for disease and mortality but rather the result of the underlying condition. Even among older adults with good health status, sleeping >8 h has been associated with higher mortality risk and the risk increased with longer sleep duration (55).

Both offspring and controls with longer duration of sleep demonstrated overall riskier metabolic profiles. Offspring and controls who slept ≥8 h had higher insulin levels and HOMA-IR and lower HDL cholesterol levels compared with their counterparts who slept less than 8 h. The definitions of short and long duration of sleep vary between studies, with short sleep duration often defined as <5 h and long sleep duration as >8 h (45). Several studies have shown a U-shape relationship between the duration of sleep and risk of T2DM (56–58), with the lowest risk among those who slept 7–8 h per night (59). Compared with 7 h of sleep, the risk for developing diabetes ranged between 1.47 and 1.95 for shorter sleep duration and between 1.4 and 3.12 for longer sleep duration (60). Other studies have shown positive association between long, but not short, sleep duration and diabetes (61, 62). A meta-analysis of seven studies found that HOMA-IR did not differ between individuals with short and long sleep durations, but what defined long and short sleep duration varied between studies (63).

Chronic low-grade inflammation has been regarded as a strong risk factor for insulin resistance in longitudinal studies, including elderly cohorts (64–66). The inflammatory biomarkers CRP and interleukin-6 (IL-6) were found to be elevated in sleep apnea and excessive sleepiness (67, 68). Therefore, the mechanism provoking diabetes in long sleepers has been hypothesized to be induced by these proinflammatory cytokines, which are elevated in chronic low-grade inflammation. A meta-analysis that included 27 studies on sleep duration concluded that long, but not short duration of sleep was associated with increased levels of CRP and IL-6 (69). Of note, the participants in these studies were mainly women and younger. In our study, sleeping for ≥8 h had been associated with insulin resistance in the offspring, but not with diabetes. We also did not find a significant difference in CRP concentrations between groups with different sleep durations. Thus, the difference in glucose metabolism in offspring and controls who were long-sleepers could not be explained by the existence of a chronic inflammatory condition.

The association of daytime napping and mortality is controversial, with studies having reported contradictory results. A large cohort study conducted in Greece has found an inverse relationship between short daytime napping and increased mortality (70). Another study conducted in Great Britain has reported an association between daytime napping and all-cause mortality, independent of preexisting health conditions (71). In a meta-analysis of 16 cohort studies, nine studies have shown an association between daytime napping and all-cause mortality (72). The largest cohort study that addressed the relationship between cardiovascular risk and napping has found a strong association between daytime napping and risk of cardiovascular mortality (73). Daytime napping for ≥30 min was associated not only with coronary artery disease but also with cancer in both genders (74). A potential explanation for the increased risk in CVD among daytime nappers may be based on the same biological mechanisms that were related to a higher incidence of MI and stroke after arousal from night sleep, which included elevated blood pressure, acute change in posture, and hypercoagulability (75, 76). We found that controls who napped regularly had higher levels of insulin and HOMA-IR. They were also more likely to have one or more of the age-related diseases compared with those who did not nap. On the other hand, offspring who napped were not found to be at increased risk for age-related diseases, suggesting that longevity genes that they have inherited from their centenarian parent may protect them from the negative impact of napping.

Melatonin, the pineal secreted hormone, has an important role in regulating the circadian rhythm and its levels have been found to decline over the lifespan (77). However, melatonin was not measured in our study. Melatonin secretion is high at night and very low during the day. Thus, an effective evaluation of melatonin secretion requires the collection of several blood samples or urine samples for measurements of its metabolite, 6-hydroxymelatonin sulfate, over a 24-h period (78, 79). Our study was not designed for repeated blood draws and all samples were collected in the morning. Future studies should consider investigating whether melatonin secretion impacts longevity.

Although our study has a number of strengths, including a cohort of relative genetic and socioeconomic homogeneity that is comprised of centenarians, offspring, and controls, it also has a number of limitations. Since all blood samples were collected at the time of study enrollment, but only a small subgroup of centenarians were questioned about their current sleep patterns, we could not associate current sleep patterns with present diseases and biochemical parameters in the centenarian group. We used a brief self-reported sleep questionnaire, as have many other published epidemiological studies (80). A correlation of 0.45 has been found between self-reported and measured sleep duration (81). However, large epidemiological studies cannot always use objectively measured techniques such as actigraphy recording. In addition, our questionnaire addressed habitual sleep patterns and did not rely on a single-day report, thereby potentially providing a more global view of sleep patterns in an individual rather than an episodic one. Since we relied on self-report of sleep patterns at age 70 in centenarians, their responses may have been subject to recall bias. Recall bias is also a consideration in offspring and controls, although to a lesser extent than in the centenarian group since the offspring and controls were asked to recall current, rather than past, sleep patterns. However, because both offspring and controls are subject to the same recall bias, the bias is non-differential among these groups and thus is unlikely to meaningfully affect the final results. Another consideration is that not all offspring may have inherited the protective longevity genes from their centenarian parent; thus, even though the offspring group is enriched for longevity genes it is likely that not everyone in that group actually possesses them. However, as a group, the offspring are enriched for longevity genes. It is also plausible that some of the controls may carry longevity genes, but that possibility is quite low given the rare phenotype of centenarians in the general population. Although our study was conducted in an Ashkenazi Jewish sample, our prior published work demonstrates that Ashkenazi Jewish centenarians are very similar to centenarians of other ethnic backgrounds (29) and our findings have been validated in several other populations. Therefore, the findings from this study may be generalizable to other ethnic groups (82, 83), but additional validation will be required.

Strong evidence exists for the association between unfavorable sleep patterns and risky metabolic profiles and cardiovascular complications in humans. However, this study demonstrated that although centenarian offspring and controls have similar sleep patterns that are associated with unfavorable metabolic profiles in both groups, only the controls were found to have higher odds of age-related diseases in the setting of napping. These findings suggest that offspring may inherit longevity genes from their long-lived parents, which protect them from the hazardous effects of unfavorable sleep patterns. Thus, the offspring and centenarians appear to be resistant to risky sleep patterns. Further prospective studies are needed to verify whether offspring of parents with exceptional longevity, who report unfavorable sleep patterns, share their parents’ protective genome.

## Ethics Statement

The study was approved by the Institutional Review Board at the Albert Einstein College of Medicine. Written informed consent was obtained from all the study participants in accordance with the Declaration of Helsinki.

## Author Contributions

LK, TG, NB, and SM contributed to the design of the study and interpretation of the data. LK and TG contributed to the acquisition of data and writing of the manuscript. TG and SM contributed to the analysis of the data. NB and SM contributed to the critical revisions of the manuscript. All authors approved the final version of the manuscript and agree to be accountable for all aspects of the work.

## Conflict of Interest Statement

The authors declare that the research was conducted in the absence of any commercial or financial relationships that could be construed as a potential conflict of interest.
